# Functions of methyltransferase-like 3 in breast cancer: pathogenesis, drug resistance, and therapeutic target

**DOI:** 10.1186/s13058-024-01869-8

**Published:** 2024-07-03

**Authors:** Dongqiong Xiao, Mingfu Zhang, Yi Qu, Xiaojuan Su

**Affiliations:** 1 Department of Pediatrics/Key Laboratory of Birth Defects and Related Diseases of Women and Children (Ministry of Education), West China Second University Hospital, Sichuan University, Chengdu, 610041 China; 2 NHC Key Laboratory of Chronobiology (Sichuan University), Chengdu, 610041 China

**Keywords:** RNA methylation, Methyltransferase-like 3, Breast cancer, Pathogenesis, Drug resistance, Treatment

## Abstract

Breast cancer (BC) is a highly prevalent malignancy worldwide, with complex pathogenesis and treatment challenges. Research reveals that methyltransferase-like 3 (METTL3) is widely involved in the pathogenesis of several tumors through methylation of its target RNAs, and its role and mechanisms in BC are also extensively studied. In this review, we aim to provide a comprehensive interpretation of available studies and elucidate the relationship between METTL3 and BC. This review suggests that high levels of METTL3 are associated with the pathogenesis, poor prognosis, and drug resistance of BC, suggesting METTL3 as a potential diagnostic or prognostic biomarker and therapeutic target. Collectively, this review provides a comprehensive understanding of how METTL3 functions through RNA methylation, which provides a valuable reference for future fundamental studies and clinical applications.

## Background

Breast cancer (BC) is a prevalent malignancy worldwide [1]. Moreover, resistance to available therapies is a major obstacle to the effective treatment of BC [2]. BC is considered a heterogeneous disease, and its pathogenesis, which is affected by genes and external circumstances, is complex and has not yet been completely clarified [1]. Therefore, insights into the mechanisms of BC occurrence and development may help improve its diagnosis, prognosis, and treatment.

Tumor formation and progression are complex processes and involve multiple mechanisms that usually require precise regulation of gene expression across diverse cells and organs [3]. N^6^-methyladenosine (m^6^A) regulates gene expression in a post-transcriptional manner, which includes the components of “writers”, “erasers”, and “readers” [4]. Writers specifically act as the catalytic subunit to add a methyl group to the N-^6^position of adenosine, including the subunit of methyltransferase-like 3/14 (METTL3/14) and Wilms’ tumor 1-associating protein (WTAP) [4]. Meanwhile, this modification is removed by erasers and is further recognized by readers such as YTHDF (YTH N^6^-methyladenosine RNA binding protein F), YTHDC (YTH domain-containing proteins), and IGF2BP (Insulin-like growth factor 2 mRNA binding protein) [4]. Recently, RNA methylation byMETTL3 has emerged as a crucial modulator of mammalian gene expression [5]. METTL3, in contrast to the structural subunit of METTL14 and WTAP in RNA m^6^A modification, has the methyltransferase function to regulate RNA stability and life-cycle events, such as splicing, translation, and degradation [6]. METTL3 also regulates the biological processes of tumor formation and progression, either as an oncogene, or by regulating other oncogenes [7]. In addition, METTL3-mediated RNA m^6^A modification induces drug resistance in cancer via epigenetic regulation of candidate drug-resistance genes [7]. Studies have especially emphasized the risk role of METTL3 in the occurrence, progression, metastasis, poor prognosis, and drug resistance of BC [8–10]. However, there is a lack of reviews that comprehensively summarize and interpret these studies.

Therefore, in this review, we comprehensively analyze all original publications on METTL3 and its roles in BC, aiming to provide a better understanding of the current status of research on METTL3 in BC.

## Functions and mechanisms of METTL3 in BC

### METTL3 contributes to the onset and progression of BC

BC is a complex disease at the cellular and molecular levels [11]. Clinical data demonstrate that METTL3 is upregulated in BC tissues, especially in phase T3-T4, or in patients with BC that are diagnosed with tumor metastasis in the lymphatic system [12]. Furthermore, findings also demonstrate the overexpression of METTL3 in BCSCs, and high expression levels of METTL3 are usually associated with a worse prognosis in patients with BC [12]. Therefore, METTL3 might be a risk factor for BC.

#### METTL3 functions by targeting oncogenes

METTL3-mediated m^6^A modification of RNAs includes coding (mRNA) and non-coding (i.e., microRNA [miRNA], long non-coding RNA [lncRNA], circular RNA [circRNA]) RNAs.

#### METTL3 and mRNA modification

BC stem cells (BCSCs) possess self-renewal and differentiation abilities to produce more BC cells, which function primarily during BC initiation, as well as play a fundamental role in BC progression, drug resistance, and recurrence [13]. Xie et al. [12] demonstrated that METTL3 modifies *SOX2* mRNA via m^6^A modification and activates BCSC stemness and malignant progression. Other studies demonstrate that by targeting *EZH2* mRNA, METTL3 promotes epithelial-mesenchymal transition (EMT) and metastasis of BCSCs, thereby aggravating the malignant phenotype of BC [14, 15]. In addition, METTL3 also increases the methylation levels, accelerates proliferation rates, promotes tumor growth, and reduces apoptosis of BC cells via m^6^A modification on *Bcl-2* mRNA [14, 15]. Xu et al. [16] reported that METTL3, together with YTHDF2, reduces large tumor suppressor kinase 1(*LTSK1*) mRNA stability, thereby promoting the proliferation and glycolytic metabolism in BC through the YAP/TAZ axis and Hippo pathways. Furthermore, alternative splicing (AS) enables differential inclusion of exons from a given transcript, thereby contributing to the transcriptome and proteome diversity [17]. Aberrant AS patterns play key roles in BC development. METTL3 functions in modulating BC-associated AS processes [18]. Specifically, both m^6^A deposition in splice site boundaries and in splicing and transcription factor transcripts, such as *MYC*, direct AS switches of specific BC-associated transcripts, which are associated with a poor overall survival rate in patients with BC, suggesting the use of these AS events as a novel potential prognostic biomarker [18].

**Fig. 1:**
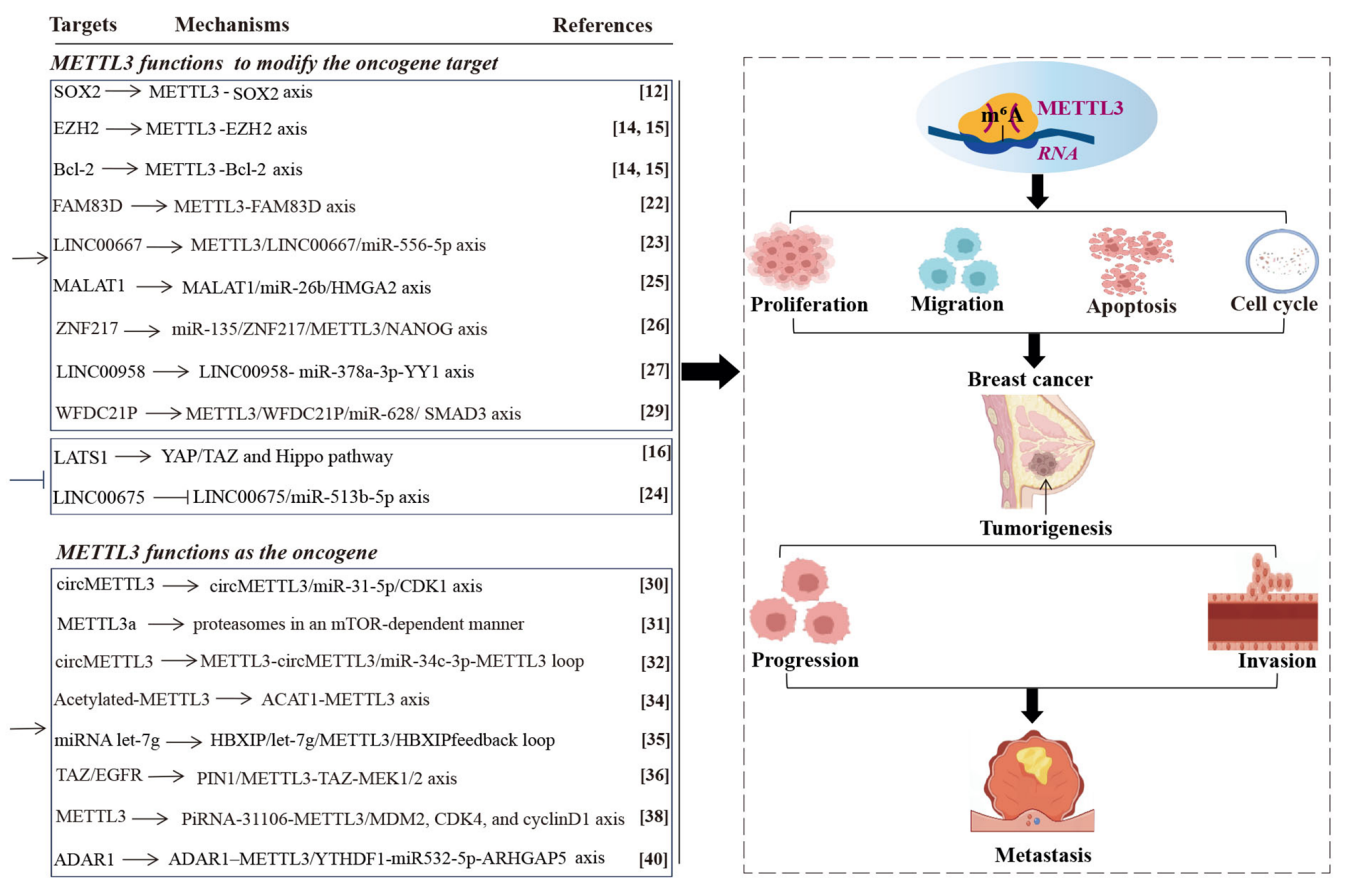
METTL3-mediated RNA m^6^A modification induces breast cancer. High levels of METTL3 are associated with the tumorigenesis, progression, invasion, and metastasis of breast cancer, which functions by mediating m^6^A modification on different RNAs, inducing the abnormal process of proliferation, migration, apoptosis, and cell cycle. Methyltransferase-like 3 (METTL3). N^6^-methyladenosine (m^6^A). Acetyl-CoA acetyltransferase 1 (ACAT1). Metastasis-associated lung adenocarcinoma transcript 1 (MALAT1). High mobility group AT-hook 2 (HMGA2). High zinc finger protein 217 (ZNF217). Large tumor suppressor kinase 1 (LATS1). Transcriptional coactivator with PDZ-binding motif (TAZ). Host gene of circular RNA METTL3 (circMETTL3). Cyclin-dependent kinase 1 (CDK1). Cleaved form of METTL3 (METTL3a). Hepatitis B X-interacting protein (HBXIP). Epidermal growth factor receptor (EGFR). Peptidyl-prolyl cis-trans isomerase NIMA-interacting 1 (PIN1). P-element-induced wimpy testis (PIWI)-interacting RNAs (piRNAs). YTH N^6^-methyladenosine RNA binding protein F1 (YTHDF1)

Triple-negative BC (TNBC) is the most dominant and malignant pathological type of BC [19]. m^6^A regulators exhibit remarkable dysregulation in TNBC tissues, whereas METTL3 shows significant downregulation [20, 21]. Moreover, the level of METTL3 is found to be tightly linked to the progression and poor survival in patients with TNBC, whereas its gene mutation and amplification are not associated with these outcomes [21]. FAM83D is markedly elevated in TNBC tissues and cells, and high-level of FAM83D is related to poor prognosis in patients with TNBC [22, 23]. Yu et al. [22] reported that METTL3 mediates m^6^A modification on *FAM838D* and upregulates FAM83D expression to activate the Wnt/β-catenin pathway, which in turn promotes TNBC malignant behaviors [22]. These findings suggest that METTL3 aggravates TNBC progression by targeting the FAM83D/Wnt/β-catenin pathway **(**Fig. 1**)**.

Collectively, these findings suggest that METTL3 functions as an oncogenic driver to modify mRNAs and triggers BC onset and progression.

#### METTL3 and non-coding RNAs

METTL3 also exerts its oncogenic function by modulating the expression of noncoding RNAs. Ren et al. [23] found that LINC00667 is an m^6^A-modified lncRNA, which is up-regulated upon KIAA1429 overexpression. The high expression of LINC00667 is correlated with the poor prognosis of patients with BC. LINC00667 promotes the proliferation and migration of BC cells. KIAA1429 targets the m^6^A modification site on LINC00667 and enhances its mRNA stability [24]. Moreover, LINC00667 positively regulates KIAA1429 via sponging miR-556-5p, forming a KIAA1429/m^6^A/LINC00667/miR-556-5p feedback loop [24]. Collectively, the central findings of this study suggest that KIAA1429-induced LINC00667 exerts functions as an oncogene in BC progression through an m^6^A-dependent feedback loop. miR-26b exhibits consistently low levels, while METTL3, metastasis-associated lung adenocarcinoma transcript 1 (MALAT1), and high mobility group AT-hook 2 (HMGA2) demonstrate elevated levels in BC [25]. METTL3 increases *MALAT1* expression by modulating its m^6^A modification, which subsequently promotes HMGA2 expression through miR-26b sponging, ultimately leading to EMT, migration, and invasion of BC cells [25]. Notably, METTL3 drives BC tumorigenesis via the MALAT1/miR-26b/HMGA2 axis.

High zinc finger protein 217 (ZNF217) expression and low miR-135 expression levels are found in BC tissues and cells [26]. Silencing ZNF217 or elevating miR-135 inhibits BC cell malignant behaviors [26]. ZNF217 upregulates METTL3 and that in turn targets NANOG to promote BC progression [26]. Therefore, the miR-135/ZNF217/METTL3/NANOG axis promotes BC progression, emphasizing METTL3 or NANOG as a potential therapeutic target for BC treatment. Rong et al. [27] showed that METTL3 facilitates LINC00958 expression by enhancing its stability in BC tissues and cells, thus revealing a novel METTL3-involving mechanism for regulating BC tumorigenesis. LINC00958 interacts with miR-378a-3p in a competitive endogenous RNA (ceRNA) manner to promote YY1 expression [27]. Therefore, BC tumor progression is further promoted and aggravated via the METTL3-LINC00958/miR-378a-3p-YY1 axis. Fan et al. [28] discovered that METTL3 downregulates LINC00675 expression, which is indicative of a higher tumor grade, worse lymphovascular invasion, and shorter survival in patients with BC. Mechanistically, LINC00675 acts as a ceRNA by interacting with miR-513b-5p and repressing its expression [28].

Furthermore, the level of LncRNA-WFDC21P is abnormally high in TNBC, which promotes TNBC cell proliferation and metastasis, as well as is strongly linked to a poor survival rate of patients. Wei et al. [29] reported that METTL3-mediated m^6^A modification on *WFDC21P* upregulates WFDC21P expression in TNBC cells, which in turn interacts with miR-628-5p and further inhibits Smad3-related gene expression to promote cell proliferation and metastasis [29]. This study demonstrates the vital roles of METTL3-mediated m^6^A modification on *WFDC21P* and its regulation in the proliferation and metastasis of TNBC cells via the METTL3/WFDC21P/miR-628/SMAD3 axis **(**Fig. 1**)**.

#### METTL3 functions as the target oncogene METTL3 functions in BC by modifying itself

In addition to regulating target gene expression, METTL3 itself is regulated by other factors in BC. Li et al. [30] revealed that *METTL3*, the host gene of circular RNA METTL3 (circMETTL3), regulates circMETTL3 expression in an m^6^A-dependent manner. However, circMETTL3 does not affect METTL3 expression. Mechanistically, circMETTL3 interacts with miR-31-5p in a ceRNA manner, thereby upregulating the expression of its target, cyclin-dependent kinase 1 (CDK1) [30]. Therefore, circMETTL3 facilitates BC cell proliferation, migration, and invasion via the circMETTL3/miR-31-5p/CDK1 axis. In addition to investigating the novel relationship between the circRNA and its corresponding host gene, another study indicated that the cleaved form of METTL3 (termed METTL3a), which is mediated by proteasomes in an mTOR-dependent manner, is essential for regulating the assembly of the METTL3-METTL14-WTAP complex [31]. Subsequently, the METTL3-METTL14-WTAP complex functions to promote BC progression by depositing m^6^A modification on RNAs, which primarily depends on its methyltransferase activity that is supported by the catalytic role of METTL3, the structural role of METTL14, as well as the adaptor role of WTAP.

In contrast, a previous study revealed a striking reduction of METTL3 levels in TNBC samples, including tissues and cell lines [32]. Functionally, METTL3 upregulates its target, circMETTL3, which consequently interacts with miR-34c-3p in a ceRNA manner [32]. Furthermore, miR-34c-3p represses METTL3 expression [32]. The negative feedback-loop regulatory mechanism of METTL3-circMETTL3/ miR-34c-3p-METTL3 promotes TNBC tumor cell proliferation, invasion, metastasis, and growth [32]. Meanwhile, a low level of METTL3 in TNBC is strongly linked to short, distant metastasis-free survival [33]. METTL3 augments migration, invasion, and adhesion of TNBC cell lines by upregulating the collagen type III alpha 1 chain, ultimately promoting the metastasis of TNBC cells [33]. In addition, Zhang et al. [34] reported an acetylation-related mechanism for TNBC progression, demonstrating that nuclear receptor subfamily 2 group F member 6 transcriptionally activates acetyl-CoA acetyltransferase 1 (*ACAT1*), which promotes TNBC cell migration and invasion via ACAT1-mediated METTL3 acetylation. Notably, although the level of METTL3 is downregulated, the modified-METTL3 (circMETTL3, METTL3 acetylation) is upregulated, which together affect the malignant processes in BC **(**Fig. 1**)**.

Collectively, these findings suggest that METTL3 contributes to BC by regulating itself, suggesting its oncogenic role. Moreover, although findings suggest a low level of METTL3 in TNBC, it functions through its modified types with the oncogenic role. Therefore, METTL3 is a risk gene for BC.

#### METTL3 functions through a regulatory network

Consistently, Cai et al. [35] reported that hepatitis B X-interacting protein (HBXIP) increases METTL3 expression in BC by suppressing miRNA let-7 g. Remarkably, METTL3-mediated m^6^A modification in turn enhances HBXIP expression [35]. Overexpression of HBXIP not only facilitates BC cell proliferation but also impairs apoptosis [35]. In summary, a positive feedback-loop regulatory mechanism, HBXIP-let-7 g/METTL3-HBXIP, is established by HBXIP to expedite BC cell proliferation. Peptidyl-prolyl cis-trans isomerase NIMA-interacting 1 (PIN1) interacts with METTL3 and thereby blocks its ubiquitin-dependent proteasomal and lysosomal degradation [36]. In clinical settings, METTL3 expression is significantly increased with tumor progression and is positively correlated with PIN1 expression in BC tissues [36]. PIN1 stabilizes METTL3, which in turn increases the m^6^A modification and translation of the transcriptional coactivator with PDZ-binding motif (*TAZ*) and the epidermal growth factor receptor (*EGFR*) mRNA [36]. Inhibition of MEK1/2 kinases and PIN1 destabilizes METTL3, which blocks BC cell proliferation and induces cell cycle arrest at the G0/G1 phases [36]. Moreover, silencing METTL3 reduces PIN1 overexpression-induced colony formation in MCF7 cells and enhances tumor growth in 4T1 cells in mouse models [36]. Therefore, PIN1 upregulates METTL3, and the PIN1/METTL3 axis may be an alternative therapeutic target for BC. P-element-induced wimpy testis (PIWI)-interacting RNAs (piRNAs) are novel non-coding RNAs whose abnormal expression is closely associated with multiple cancers [37]. Huang et al. [38] reported that the level of PiRNA*-31,106* is high in BC tissues and cell lines (MDA-MB-231 and MCF-7), which promotes the m^6^A methylation levels and facilitates METTL3 expression that further promotes BC progression by upregulating the expressions of MDM2, CDK4, and cyclinD1 [38]. Therefore, *PiRNA-31,106* upregulates METTL3 to promote BC progression, suggesting its potential as a therapeutic target in BC **(**Fig. 1**)**.

To sum up, these findings highlight that METTL3 regulates BC progression through various mechanisms, including interacting with other molecules and influencing multiple pathways. Therefore, while METTL3 does have a direct impact on oncogenic processes, its role is also mediated through complex regulatory networks.

#### METTL3 functions by interacting with A-to-I RNA editing

Furthermore, A-to-I RNA editing is another prevalent type of RNA modification that controls gene expression in mammals and regulates tumorigenesis and tumor progression [39]. Specifically, ADAR1 is an A-to-I RNA-editing enzyme, both ADAR1 and METTL3 are upregulated in BC samples [40]. Li et al. [40] showed that ADAR1 interacts with METTL3 in a YTHDF1-dependent manner and further increases METTL3 expression. Mechanically, ADAR1 changes the binding site of *METTL3* mRNA on miR532-5p and is recognized by YTHDF1. Subsequently, METTL3 expression is increased, which further targets *ARHGAP5* to promote the proliferation, migration, and invasion of BC cells [40]. Loss of ADAR1 or METTL3 significantly inhibits BC growth in vivo [40]. Notably, the ADAR1-METTL3 axis is a novel pathway that connects the RNA regulation mechanism of A-to-I editing and METTL3-mediated m^6^A modification during BC progression. Therefore, the combined targeting of ADAR1 or METTL3 might be more effective for BC treatment **(**Fig. 1**)**.

Collectively, these findings suggest that METTL3 is primarily associated with BC initiation and progression, which functions by regulating the oncogene target or functions as the target oncogene. Furthermore, these findings suggest that METTL3 has great clinical potential as a diagnosis biomarker.

### METTL3 induces drug resistance and poor prognosis of BC

Chemotherapy is an important strategy for BC treatment [41]. *Adriamycin* (ADR), *Tamoxifen*, and *Paclitaxel* are currently considered the primary therapeutic drugs for patients with BC [42]. However, clinical data suggest that the majority of patients with BC experience resistance to chemotherapy [41]. Specifically, resistance to ADR and *Tamoxifen* is considered a troublesome challenge for curing patients with BC [43]. Currently, studies have also suggested the implication of METTL3-mediated RNA m^6^A modification in the development of BC chemoresistance. The global levels of mRNA m^6^A methylation and METTL3 are upregulated in ADR-resistant BC cell lines while silencing METTL3 partially overcomes this resistance [44]. METTL3 cooperates with IGF2BP3 to modulate the m^6^A modification on *HYOU1* mRNA and increases *HYOU1* stability, which subsequently increases ADR resistance in BC cells [44]. Therefore, the METTL3/IGF2BP3-HYOU1 axis modulates ADR sensitivity in BC cells, targeting this axis might be a strategy to improve ADR efficacy in BC treatment.

Zhou et al. [43] demonstrated that METTL3-mediated m^6^A modification on long noncoding RNA (lncRNA) KCNQ1OT1 promotes ADR resistance in BC by regulating the miR-103a-3p/multidrug resistance protein 1 (MDR1) axis. LncRNA KCNQ1OT1 is highly expressed in ADR-resistant BC cells, and its expression is modulated by MELLT3 via m^6^A modification. A further mechanistic study found that miR-103a-3p acts as a sponge for lncRNA KCNQ1OT1 and MDR1 [43]. Altogether, these findings indicate that the METTL3-lncRNAKCNQ1OT1/ miR-103a-3p-MDR1 axis is responsible for ADR resistance in BC, which suggests a novel approach to prevent ADR resistance in patients with BC. Consistently, high levels of METTL3 enhance the ADR resistance for BC cells by upregulating miR-221-3p through positive regulation of homeodomain-interacting protein kinase 2 (HIPK2) and its direct target *Che-1* [45]. More detailed, METTL3 functions to promote miR-221-3p expression by decreasing m^6^A mRNA methylation on pri-miR-221-3p [45]. Subsequently, miR-221-3p further enhances the expression of MDR1 and BCRP, which promotes a higher production of ADR-resistant cells while inhibiting apoptosis in such cells [45]. Together, after the cooperation of these signaling molecules, the METTL3-HIPK2/Che-1-miR-221-3p-MDR1/BCRP axis triggers ADR resistance in BC cells. Li et al. [46] found that METTL3 is highly expressed in ADR-resistant BC cells and upregulates *MALAT1* through m^6^A modification, which, in turn, recruits E2F transcription factor 1(E2F1) to activate anterior gradient 2 (AGR2) transcription and increases ADR resistance in BC cells. Collectively, METTL3 promotes ADR resistance in BC by targeting the MALAT1/E2F1/AGR2 axis. Moreover, Li et al. [47] reported that METTL3 promotes the efficiency of homologous recombination repair and inhibits ADR-induced DNA damage. METTL3 functions by enhancing epidermal growth factor (EGF) synthesis in a YTHDC1-dependent manner, and EGF subsequently inhibits RAD51 expression [47]. These findings reveal the mechanism of METTL3 in regulating ADR resistance in BC by targeting the EGF/RAD51 axis. Furthermore, Liu et al. [48] revealed that the expression of METTL3 and adenylate kinase 4 (AK4) is remarkably elevated in *Tamoxifen*-resistant BC cells compared with that in their parental counterparts. Further investigation found that METTL3 methylates *AK4* mRNA on the 5′-untranslated region, leading to *Tamoxifen* resistance in patients with BC [46].

**Fig. 2:**
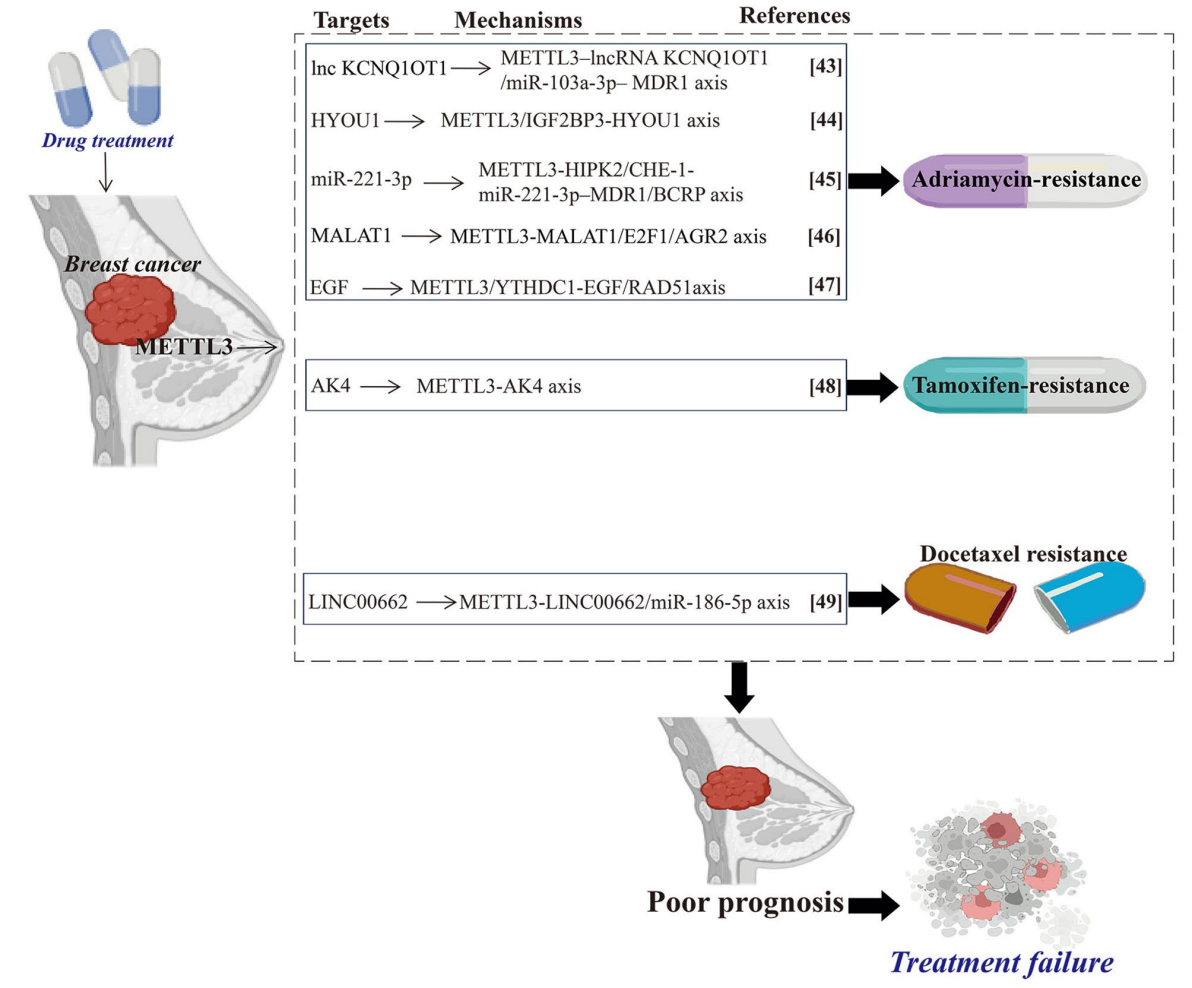
METTL3 contributes to the drug resistance of breast cancer. High levels of METTL3 are associated with drug resistance and poor prognosis of breast cancer by mediating m^6^A modification on different RNAs. Methyltransferase-like 3 (METTL3). N^6^-methyladenosine (m^6^A). Multidrug resistance protein 1 (MDR1). Homeodomain-interacting protein kinase 2 (HIPK2). Metastasis-associated lung adenocarcinoma transcript 1 (MALAT1). E2F transcription factor 1(E2F1). Anterior gradient 2 (AGR2). Epidermal growth factor (EGF). YTH domain containing 1 (YTHDC1). Adenylate kinase 4 (AK4)

Consistently, Jing et al. [49] reported that METTL3 regulates *Docetaxel* resistance of TNBC cells by regulating a feedback loop. They found that METTL3 uses LINC00662 as its target, which directly interacts with miR-186-5p, and METTL3 expression is in turn regulated by miR-186-5p [49]. LINC00662 and miR-186-5p regulate the viability of *Docetaxel*-resistant BC cells, and miR-186-5p subsequently regulates METTL3 expression [49]. Overall, these findings suggest that suppression of METTL3 promotes *Docetaxel* resistance in BC through the METTL3-LINC00662/miR‑186‑5p-METTL3 signaling **(**Fig. 2**)**.

However, METTL3 knockdown decreases the drug sensitivity of hormone receptor-positive/human epidermal growth factor receptor 2-negative (HR + HER2^−^) cells by targeting *CDKN1A*, activating the AKT pathway, and promoting EMT [50]. Moreover, METTL3 knockdown also leads to the inhibition of BAX and caspase-3/-9/-8 [50]. Therefore, METTL3 might play a tumor-suppressor role and it could be a potential biomarker for predicting the prognosis of patients with HR + HER2-BC.

Collectively, these data suggest that METTL3 contributes to various drug resistance of BC, which usually indicates a poor prognosis and recurrence of BC. Therefore, we suppose that METTL3 is a promising prognostic biomarker for BC.

### Targeting METTL3 for BC therapy

Considering that a high level of METTL3 promotes BC occurrence, progression, and drug resistance. Therefore, inhibition of METTL3 is considered a potential approach for promoting chemotherapeutic effectiveness against BC. STM2457, the small-molecule inhibitor of METTL3, significantly reduces the proliferation, viability, and migration of TNBC cells, including both *BRCA1/2* wild-type and *BRCA1-*mutated cell lines [51]. Therefore, STM2457 displays anti-tumor activity in TNBC and enhances chemotherapy response while sensitizing *BRCA1/2* wildtype patients to *Olaparib*.

Epigenetic drugs are a novel therapeutic method for BC [52]. *Cromolyn* is a mast cell stabilizer emerging as an anticancer drug [53]. The study suggests that chitosan nanoparticle encapsulated *Cromolyn* shows a prominent anticancer effect in MCF-7 cells by reducing the cell viability percent and enhancing DNA damage [54]. Mechanistically, it functions by inhibiting DNA methyltransferase 1 (DNMT1) and METTL3, as well as reversing the hypermethylation pattern of the tumor suppressor genes *RASSF1A* and *p16* [54]. In addition, it diminishes ERK1/2 phosphorylation, which affects DNMT1 expression [54]. Moreover, in vivo, it lessens the tumor volume and halts DNMT1 and METTL3 expression in BC mice [54]. Therefore, chitosan nanoparticle encapsulated *Cromolyn* acts as an epigenetic drug by inhibiting ERK1/2 phosphorylation/DNMT1/DNA methylation and impacting the RNA methylation via inhibiting METTL3 expression. Additionally, Cheng et al. [55] reported that *Metformin* inhibits BC progression by reducing METTL3 expression mediated by miR-483-3p. In detail, the inhibited expression of METTL3 reduces BC cell proliferation by modulating p21 expression in an m^6^A-dependent manner [55]. These findings suggest that *Metformin* functions to relieve BC by inhibiting METTL3 via p21, suggesting METTL3 is a potential therapeutic target for BC treatment. Furthermore, Wan et al. [56] reported that the inhibition of METTL3 enhances tumor immune surveillance. Mechanistically, the downregulated expression of METTL3 cooperates with IGF2BP3 to reduce the m^6^A modification and the expression of programmed cell death 1 ligand 1 (PDL-1), which subsequently heightens antitumor immunity through PDL-1- mediated T-cell activation, exhaustion, and infiltration [56]. These findings suggest that inhibition of METTL3 is an effective strategy for BC immunotherapy.

## Conclusions

The high incidence and mortality of BC have been the major health problems worldwide for the last few decades [57]. Extensive studies have identified the mechanisms underlying BC pathogenesis and therapeutic failure, which are primarily a result of alterations in oncogenes and tumor suppressor genes, leading to protein dysregulation and carcinogen activation [58]. Therefore, studies on epigenetic modifications underlying the occurrence, progression, and metastasis of BC have attracted great attention. METTL3 has the catalytic ability to regulate the biological characteristics of RNA (i.e., stability and translation) [6]. METTL3 also accelerates the proliferation, migration, and invasion of BC cells via posttranscriptional modifications. Herein, we summarize all original publications on this topic and classify the findings into different categories to further analyze and interpret the roles, mechanisms, and relationships of METTL3 during different events in BC, including occurrence and progression, treatment failure, as well as targeting METTL3 for therapy.

This review suggests that high levels of METTL3 act as an oncogene by targeting mRNAs and noncoding RNAs during the occurrence and progression of BC, and increase chemoresistance. In addition to regulating target gene expression, METTL3 functions as a target gene for other risk factors and regulators in BC. The underlying mechanism of METTL3 functions includes inducing the abnormal process of proliferation, migration, apoptosis, and cell cycle by mediating m^6^A modification on different RNAs. Furthermore, data also hint to us that targeting METTL3 is a promising strategy for BC treatment. In summary, these findings suggest that METTL3 with great clinical potential as a diagnostic and prognostic biomarker, as well as a treatment target.

Although the analysis of this review suggests METTL3 is a risk gene for BC, contrast findings are also found in patients with HR + HER2-BC, which suggest a tumor suppressor role for METTL3. However, due to the only one available publications on the role of METTL3 in patients with HR + HER2-BC, the conclusion for METTL3’s role in HR + HER2-BC needs further investigation. Besides, current available methods for downregulating METTL3 include the small-molecule inhibitor STM2457, epigenetic drugs *Cromolyn* and *Metformin*, as well as PDL-1. However, to our knowledge, preclinical studies for treating patients with BC by targeting METTL3 are not yet available. Furthermore, combination therapies involving METTL3 inhibitors and other methods have not been reported as well. To sum up, these are the main limitations of the existing evidence supporting METTL3’s role in BC. Therefore, we suppose that future studies and clinical validation are strongly required to bridge such gaps to improve the understanding of METTL3’ role in BC.

Collectively, this review provides a systematic interpretation of the risk role of METTL3 in BC, which provides a convenient reference for exploring its clinical potential in the future.

## Data Availability

No datasets were generated or analysed during the current study.

## Abbreviations

BC: Breast cancer
METTL3: Methyltransferase-like 3
m^6^A: N^6^-methyladenosine
BCSCs: BC stem cells
EMT: Epithelial-mesenchymal transition
YTHDF: YTH N^6^-methyladenosine RNA binding protein F
LTSK1: Large tumor suppressor kinase 1
AS: Alternative splicing
MALAT1: Metastasis-associated lung adenocarcinoma transcript 1
HMGA2: High mobility group AT-hook 2
ZNF217: High zinc finger protein 217
ceRNA: Competitive endogenous RNA
circMETTL3: Host gene of circular RNA METTL3
CDK1: Cyclin-dependent kinase 1
METTL3a: Cleaved form of METTL3
HBXIP: Hepatitis B X-interacting protein
PIN1: Peptidyl-prolyl cis-trans isomerase NIMA-interacting 1
TAZ: Transcriptional coactivator with PDZ-binding motif
EGFR: Epidermal growth factor receptor
piRNAs: P-element-induced wimpy testis (PIWI)-interacting RNAs
TNBC: Triple-negative BC
ACAT1: Acetyl-CoA acetyltransferase 1
ADR: Adriamycin
IGF2BP3: Insulin-like growth factor 2 mRNA binding protein 3
lncRNA: Long noncoding RNA
MDR1: Multidrug resistance protein 1
HIPK2: Homeodomain-interacting protein kinase 2
E2F1: E2F transcription factor 1
AGR2: Anterior gradient 2
EGF: Epidermal growth factor
AK4: Adenylate kinase 4
DNMT1: DNA methyltransferase 1
PDL-1: Programmed cell death 1 ligand 1
